# Naphthalene-1,4,5,8-tetra­carboxylic acid 1,8-anhydride–4,4′-bipyridine (1/1)

**DOI:** 10.1107/S1600536809044146

**Published:** 2009-10-31

**Authors:** Ji-Hua Deng, Meng-Ping Guo, Qiao-Chu Zhang, Lin Yuan, Hui-Rui Guo, Guang-Quan Mei

**Affiliations:** a College of Chemistry & Bio-engineering, Yichun University, Yichun, Jiangxi 336000, People’s Republic of China; b Institute of Coordination catalysis, Yichun University, Yichun, Jiangxi 336000, People’s Republic of China

## Abstract

The title compound, C_14_H_6_O_7_·C_10_H_8_N_2_, has been hydro­thermally synthesized. Structural ananlysis indicates that the crystals are produced by cocrystallization of naphthalene-1,4,5,8-tetra­carboxylic acid 1,8-anhydride and 4,4′-bipyridine (bpy) mol­ecules. The crystal packing is stabilized by inter­molecular O—H⋯N and C—H⋯O hydrogen bonds and π–π stacking inter­actions [centroid–centroid distances = 3.5846 (9) Å].

## Related literature

For the structures of naphthalene-1,4,5,8-tetra­carboxylic acid 1,8-anhydride, its DMSO solvate and several metal complexes, see: Blackburn *et al.* (1997 ▶); Fitzgerald *et al.* (1992 ▶); Robl (1987 ▶); Xu *et al.* (2005*a* ▶,*b* ▶.) For hydrogen bonds, see: Desiraju & Steiner (1999 ▶).
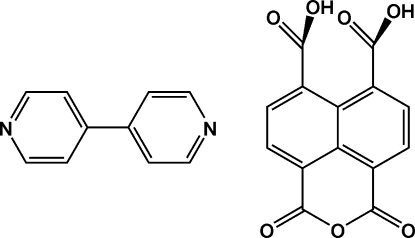


## Experimental

### 

#### Crystal data

C_14_H_6_O_7_·C_10_H_8_N_2_
*M* *_r_* = 442.37
Triclinic, 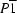

*a* = 9.6193 (8) Å
*b* = 9.6964 (3) Å
*c* = 10.192 (1) Å
α = 81.384 (5)°
β = 85.615 (6)°
γ = 83.947 (3)°
*V* = 932.9 (1) Å^3^
*Z* = 2
Mo *K*α radiation
μ = 0.12 mm^−1^
*T* = 173 K
0.28 × 0.24 × 0.20 mm

#### Data collection

Bruker SMART CCD area-detector diffractometer
Absorption correction: multi-scan (*SADABS*; Bruker, 1998 ▶) *T*_min_ = 0.968, *T*_max_ = 0.977
6648 measured reflections
3240 independent reflections
2772 reflections with *I* > 2σ(*I*)
*R*_int_ = 0.017

#### Refinement

*R*[*F* ^2^ > 2σ(*F* ^2^)] = 0.033
*wR*(*F* ^2^) = 0.104
*S* = 1.06
3240 reflections
300 parameters
H-atom parameters constrained
Δρ_max_ = 0.21 e Å^−3^
Δρ_min_ = −0.21 e Å^−3^

### 

Data collection: *SMART* (Bruker, 1998 ▶); cell refinement: *SAINT* (Bruker, 1998 ▶); data reduction: *SAINT*; program(s) used to solve structure: *SHELXS97* (Sheldrick, 2008 ▶); program(s) used to refine structure: *SHELXL97* (Sheldrick, 2008 ▶); molecular graphics: *SHELXTL* (Sheldrick, 2008 ▶); software used to prepare material for publication: *SHELXTL*.

## Supplementary Material

Crystal structure: contains datablocks I, global. DOI: 10.1107/S1600536809044146/im2151sup1.cif

Structure factors: contains datablocks I. DOI: 10.1107/S1600536809044146/im2151Isup2.hkl

Additional supplementary materials:  crystallographic information; 3D view; checkCIF report

## Figures and Tables

**Table 1:** Hydrogen-bond geometry (Å, °)

*D*—H⋯*A*	*D*—H	H⋯*A*	*D*⋯*A*	*D*—H⋯*A*
O2—H2⋯N2^i^	0.84	1.77	2.594 (2)	167
O4—H4*A*⋯N1^ii^	0.84	1.74	2.573 (2)	171
C16—H16⋯O1^iii^	0.95	2.36	3.254 (2)	157
C22—H22⋯O3^iv^	0.95	2.57	3.425 (2)	150

Symmetry codes: (i) 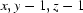; (ii) 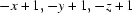; (iii) 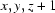; (iv) 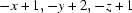.

**Fig. 1.**
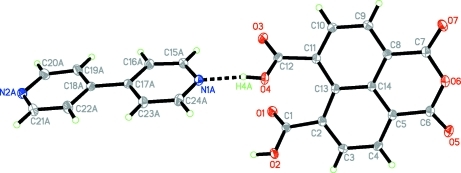
The crystal structure of (I), Symmetry code: -x + 1, -y + 1, -z + 1.

**Fig. 2.**
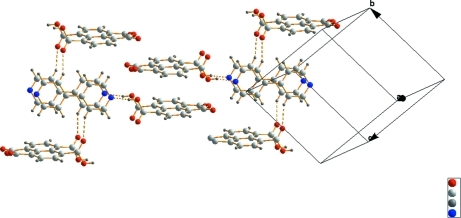
The intermolecular hydrogen bonds (dashed lines) and π-π stacking interactions existing in the crystal structure of (I).

## supplementary crystallographic information

### Comment

Several crystal structures of naphthalene-1,4,5,8-tetracarboxylic acid 1,8-anhydride (ntaa) (Xu *et al.*, 2005*b*), its DMSO solvate (Blackburn *et al.*, 1997) and some metal complexes (Fitzgerald *et al.*, 1992; Robl *et al.*, 1987; Xu *et al.*, 2005*a*) have been reported in the literature. Herein, we report the synthesis and crystal crystal structure of a new compound of ntaa.

The structure of the title compound, (I), consists of one ntaa molecule and one bpy molecule. The two carboxylate groups of the ntaa are not coplanar with the naphthalene ring. The corresponding dihedral angles O1-C1-C2-C13 and O3-C12-C11-C10 are 50.8 (2)° and 52.7 (2)°, respectively. The two pyridyl rings of the bpy molecule are almost coplanar with a dihedral angle of 3.6 (3)° (Fig. 1).

The molecules are held together by intermolecular hydrogen bonding interactions (Desiraju *et al.* 1999) and π-π stacking interactions, forming a three-dimensional supramolecular network. Two O–H···N hydrogen bonds with O···O distances of 2.594 (2) and 2.573 (2) Å are formed with the two carboxylic acid OH groups as donors and the N atoms of the two inequivalent bpy molecules as acceptors (Table 1, Fig. 1 and Fig. 2). In addition, π-π stacking interactions between two pyridyl rings (3.346 Å) and two naphthalene rings (3.357 Å), are also observed.

### Experimental

A mixture of 0.5 mmol NiCl_2_ × 6 H_2_O, 0.5 mmol of naphthalene-1,4,5,8-tetracarboxylic acid, 0.5 mmol of 4,4-bipyridine, 1.0 mmol of NaOH and 10 ml distilled water was heated to 383 K for six days in a 20 ml sealed Teflon-lined stainless steel vessel. After the autoclave was cooled to room temperature, block-shaped yellow crystals of (I) were isolated by filtration, washed with water, and dried in air. (yield: 53.2% based on naphthalene-1,4,5,8-tetracarboxylic acid)

### Refinement

Hydrogen atoms attached to carbon and oxygen atoms were positioned geometrically and treated as riding, with C—H = 0.95 Å, O—H = 0.84 Å, and *U*_iso_(H) = 1.2*U*_eq_(C), *U*_iso_(H) = 1.5*U*_eq_(O).

### Crystal data

**Table:**

C_14_H_6_O_7_·C_10_H_8_N_2_	*Z* = 2
*M**_r_* = 442.37	*F*(000) = 456
Triclinic, *P*1	*D*_x_ = 1.575 Mg m^−^^3^
Hall symbol: -P 1	Mo *K*α radiation, λ = 0.71073 Å
*a* = 9.6193 (8) Å	Cell parameters from 5003 reflections
*b* = 9.6964 (3) Å	θ = 2.9–27.1°
*c* = 10.192 (1) Å	µ = 0.12 mm^−^^1^
α = 81.384 (5)°	*T* = 173 K
β = 85.615 (6)°	Block, yellow
γ = 83.947 (3)°	0.28 × 0.24 × 0.20 mm
*V* = 932.9 (1) Å^3^	

### Data collection

**Table:**

Bruker SMART CCD area-detector diffractometer	3240 independent reflections
Radiation source: fine-focus sealed tube	2772 reflections with *I* > 2σ(*I*)
graphite	*R*_int_ = 0.017
φ and ω scans	θ_max_ = 25.0°, θ_min_ = 2.0°
Absorption correction: multi-scan (*SADABS*; Bruker, 1998\bbr00)	*h* = −11→11
*T*_min_ = 0.968, *T*_max_ = 0.977	*k* = −11→11
6648 measured reflections	*l* = −12→12

### Refinement

**Table:**

Refinement on *F*^2^	Primary atom site location: structure-invariant direct methods
Least-squares matrix: full	Secondary atom site location: difference Fourier map
*R*[*F*^2^ > 2σ(*F*^2^)] = 0.033	Hydrogen site location: inferred from neighbouring sites
*wR*(*F*^2^) = 0.104	H-atom parameters constrained
*S* = 1.06	*w* = 1/[σ^2^(*F*_o_^2^) + (0.0664*P*)^2^ + 0.1626*P*] where *P* = (*F*_o_^2^ + 2*F*_c_^2^)/3
3240 reflections	(Δ/σ)_max_ < 0.001
300 parameters	Δρ_max_ = 0.21 e Å^−^^3^
0 restraints	Δρ_min_ = −0.21 e Å^−^^3^

### Special details

**Table:**

Geometry. All e.s.d.'s (except the e.s.d. in the dihedral angle between two l.s. planes) are estimated using the full covariance matrix. The cell e.s.d.'s are taken into account individually in the estimation of e.s.d.'s in distances, angles and torsion angles; correlations between e.s.d.'s in cell parameters are only used when they are defined by crystal symmetry. An approximate (isotropic) treatment of cell e.s.d.'s is used for estimating e.s.d.'s involving l.s. planes.
Refinement. Refinement of *F*^2^ against ALL reflections. The weighted *R*-factor *wR* and goodness of fit *S* are based on *F*^2^, conventional *R*-factors *R* are based on *F*, with *F* set to zero for negative *F*^2^. The threshold expression of *F*^2^ > σ(*F*^2^) is used only for calculating *R*-factors(gt) *etc*. and is not relevant to the choice of reflections for refinement. *R*-factors based on *F*^2^ are statistically about twice as large as those based on *F*, and *R*- factors based on ALL data will be even larger.

### Fractional atomic coordinates and isotropic or equivalent isotropic displacement parameters (Å^2^)

**Table:**

	*x*	*y*	*z*	*U*_iso_*/*U*_eq_	
O6	1.12369 (10)	0.85984 (10)	0.57565 (9)	0.0273 (2)	
O3	0.55296 (10)	0.61928 (11)	0.24288 (10)	0.0327 (3)	
O7	0.92667 (10)	0.94482 (10)	0.67238 (10)	0.0299 (3)	
O1	0.83856 (10)	0.51984 (10)	0.08988 (9)	0.0296 (2)	
O5	1.31804 (10)	0.81386 (10)	0.45444 (10)	0.0304 (3)	
C8	0.90124 (14)	0.79831 (13)	0.50961 (13)	0.0228 (3)	
C14	0.97444 (13)	0.70939 (13)	0.42367 (13)	0.0207 (3)	
C13	0.89868 (13)	0.63253 (13)	0.34836 (12)	0.0207 (3)	
C11	0.74906 (14)	0.65192 (13)	0.36207 (13)	0.0226 (3)	
C2	0.98025 (14)	0.54118 (13)	0.26762 (13)	0.0219 (3)	
C5	1.12245 (14)	0.69889 (13)	0.41277 (13)	0.0230 (3)	
C10	0.68213 (14)	0.74534 (14)	0.44146 (14)	0.0262 (3)	
H10	0.5828	0.7608	0.4452	0.031*	
C7	0.97886 (14)	0.87102 (13)	0.59342 (13)	0.0243 (3)	
C6	1.19789 (14)	0.79167 (14)	0.47810 (13)	0.0246 (3)	
C12	0.65343 (14)	0.56573 (14)	0.30445 (13)	0.0241 (3)	
C4	1.19675 (14)	0.60634 (14)	0.33777 (14)	0.0261 (3)	
H4	1.2962	0.5972	0.3331	0.031*	
C1	0.91902 (14)	0.46243 (14)	0.17226 (13)	0.0229 (3)	
C9	0.75773 (15)	0.81770 (14)	0.51654 (14)	0.0265 (3)	
H9	0.7098	0.8800	0.5721	0.032*	
C3	1.12416 (14)	0.52589 (14)	0.26854 (14)	0.0257 (3)	
H3	1.1757	0.4585	0.2205	0.031*	
N1	0.46466 (12)	0.72761 (13)	0.75937 (12)	0.0280 (3)	
N2	0.84743 (12)	1.16076 (13)	1.06975 (12)	0.0298 (3)	
C17	0.62220 (13)	0.89707 (14)	0.87676 (13)	0.0228 (3)	
C18	0.70313 (13)	0.98835 (14)	0.94187 (13)	0.0227 (3)	
C23	0.52433 (15)	0.95430 (15)	0.78427 (14)	0.0272 (3)	
H23	0.5101	1.0529	0.7596	0.033*	
C22	0.68533 (16)	1.13357 (15)	0.91138 (14)	0.0305 (3)	
H22	0.6234	1.1769	0.8457	0.037*	
C15	0.55791 (15)	0.67228 (15)	0.84780 (15)	0.0310 (3)	
H15	0.5694	0.5733	0.8706	0.037*	
C24	0.44849 (15)	0.86730 (15)	0.72895 (14)	0.0297 (3)	
H24	0.3820	0.9081	0.6667	0.036*	
C16	0.63843 (15)	0.75167 (15)	0.90771 (15)	0.0302 (3)	
H16	0.7043	0.7076	0.9694	0.036*	
C21	0.75874 (16)	1.21431 (15)	0.97767 (14)	0.0310 (3)	
H21	0.7449	1.3133	0.9561	0.037*	
C20	0.86494 (16)	1.02078 (16)	1.10009 (16)	0.0362 (4)	
H20	0.9275	0.9808	1.1663	0.043*	
C19	0.79578 (16)	0.93242 (16)	1.03902 (15)	0.0331 (3)	
H19	0.8113	0.8338	1.0632	0.040*	
O4	0.68631 (10)	0.43151 (10)	0.33780 (10)	0.0283 (2)	
H4A	0.6303	0.3865	0.3059	0.042*	
O2	0.96983 (10)	0.33081 (10)	0.18631 (10)	0.0280 (2)	
H2	0.9261	0.2867	0.1405	0.042*	

### Atomic displacement parameters (Å^2^)

**Table:**

	*U*^11^	*U*^22^	*U*^33^	*U*^12^	*U*^13^	*U*^23^
O6	0.0303 (5)	0.0273 (5)	0.0267 (5)	−0.0074 (4)	−0.0067 (4)	−0.0057 (4)
O3	0.0249 (5)	0.0356 (6)	0.0398 (6)	0.0001 (4)	−0.0124 (4)	−0.0093 (5)
O7	0.0389 (6)	0.0248 (5)	0.0279 (5)	0.0009 (4)	−0.0085 (4)	−0.0093 (4)
O1	0.0328 (6)	0.0302 (5)	0.0268 (5)	−0.0008 (4)	−0.0087 (4)	−0.0050 (4)
O5	0.0256 (5)	0.0315 (5)	0.0358 (6)	−0.0100 (4)	−0.0071 (4)	−0.0028 (4)
C8	0.0273 (7)	0.0182 (6)	0.0232 (7)	−0.0038 (5)	−0.0032 (5)	−0.0020 (5)
C14	0.0237 (7)	0.0173 (6)	0.0213 (6)	−0.0036 (5)	−0.0038 (5)	−0.0005 (5)
C13	0.0232 (7)	0.0184 (6)	0.0206 (6)	−0.0039 (5)	−0.0043 (5)	−0.0003 (5)
C11	0.0235 (7)	0.0208 (6)	0.0234 (7)	−0.0028 (5)	−0.0041 (5)	−0.0012 (5)
C2	0.0247 (7)	0.0195 (6)	0.0217 (6)	−0.0043 (5)	−0.0027 (5)	−0.0010 (5)
C5	0.0247 (7)	0.0208 (6)	0.0236 (7)	−0.0050 (5)	−0.0050 (5)	0.0002 (5)
C10	0.0207 (7)	0.0262 (7)	0.0320 (8)	−0.0013 (5)	−0.0025 (6)	−0.0056 (6)
C7	0.0289 (7)	0.0194 (6)	0.0246 (7)	−0.0027 (5)	−0.0064 (6)	−0.0007 (5)
C6	0.0274 (8)	0.0217 (6)	0.0243 (7)	−0.0038 (5)	−0.0068 (6)	0.0013 (5)
C12	0.0203 (7)	0.0285 (7)	0.0244 (7)	−0.0039 (5)	−0.0010 (5)	−0.0054 (6)
C4	0.0191 (7)	0.0277 (7)	0.0318 (7)	−0.0039 (5)	−0.0024 (6)	−0.0036 (6)
C1	0.0230 (7)	0.0239 (7)	0.0224 (7)	−0.0045 (5)	0.0001 (5)	−0.0042 (5)
C9	0.0279 (7)	0.0239 (7)	0.0282 (7)	0.0002 (5)	−0.0007 (6)	−0.0076 (6)
C3	0.0239 (7)	0.0252 (7)	0.0286 (7)	−0.0013 (5)	−0.0001 (6)	−0.0071 (6)
N1	0.0241 (6)	0.0320 (7)	0.0308 (6)	−0.0072 (5)	−0.0007 (5)	−0.0112 (5)
N2	0.0283 (6)	0.0332 (7)	0.0314 (7)	−0.0050 (5)	−0.0024 (5)	−0.0141 (5)
C17	0.0203 (7)	0.0275 (7)	0.0218 (7)	−0.0029 (5)	0.0012 (5)	−0.0082 (5)
C18	0.0205 (7)	0.0274 (7)	0.0217 (7)	−0.0027 (5)	0.0008 (5)	−0.0086 (5)
C23	0.0296 (7)	0.0257 (7)	0.0273 (7)	−0.0055 (6)	−0.0055 (6)	−0.0032 (6)
C22	0.0365 (8)	0.0295 (7)	0.0270 (7)	−0.0056 (6)	−0.0094 (6)	−0.0033 (6)
C15	0.0279 (7)	0.0253 (7)	0.0416 (8)	−0.0015 (6)	−0.0049 (6)	−0.0105 (6)
C24	0.0279 (7)	0.0350 (8)	0.0280 (7)	−0.0072 (6)	−0.0060 (6)	−0.0052 (6)
C16	0.0267 (7)	0.0278 (7)	0.0374 (8)	−0.0002 (6)	−0.0101 (6)	−0.0067 (6)
C21	0.0391 (8)	0.0258 (7)	0.0296 (8)	−0.0083 (6)	−0.0029 (6)	−0.0056 (6)
C20	0.0358 (8)	0.0341 (8)	0.0423 (9)	0.0034 (6)	−0.0161 (7)	−0.0144 (7)
C19	0.0363 (8)	0.0268 (7)	0.0388 (8)	0.0013 (6)	−0.0136 (7)	−0.0103 (6)
O4	0.0290 (5)	0.0247 (5)	0.0337 (6)	−0.0094 (4)	−0.0100 (4)	−0.0037 (4)
O2	0.0312 (5)	0.0230 (5)	0.0329 (6)	−0.0025 (4)	−0.0097 (4)	−0.0100 (4)

### Geometric parameters (Å, °)

**Table:**

O6—C7	1.3859 (17)	C9—H9	0.9500
O6—C6	1.3870 (17)	C3—H3	0.9500
O3—C12	1.2193 (16)	N1—C15	1.3349 (19)
O7—C7	1.2028 (16)	N1—C24	1.3387 (19)
O1—C1	1.2175 (16)	N2—C21	1.3271 (19)
O5—C6	1.1977 (16)	N2—C20	1.342 (2)
C8—C9	1.3713 (19)	C17—C16	1.3926 (19)
C8—C14	1.4179 (19)	C17—C23	1.3970 (19)
C8—C7	1.4742 (18)	C17—C18	1.4933 (18)
C14—C5	1.4136 (19)	C18—C22	1.391 (2)
C14—C13	1.4308 (18)	C18—C19	1.393 (2)
C13—C11	1.4305 (18)	C23—C24	1.3772 (19)
C13—C2	1.4319 (19)	C23—H23	0.9500
C11—C10	1.3808 (19)	C22—C21	1.384 (2)
C11—C12	1.5105 (18)	C22—H22	0.9500
C2—C3	1.3771 (19)	C15—C16	1.380 (2)
C2—C1	1.5098 (18)	C15—H15	0.9500
C5—C4	1.374 (2)	C24—H24	0.9500
C5—C6	1.4732 (18)	C16—H16	0.9500
C10—C9	1.4001 (19)	C21—H21	0.9500
C10—H10	0.9500	C20—C19	1.381 (2)
C12—O4	1.3064 (16)	C20—H20	0.9500
C4—C3	1.3961 (19)	C19—H19	0.9500
C4—H4	0.9500	O4—H4A	0.8400
C1—O2	1.3086 (16)	O2—H2	0.8400
			
C7—O6—C6	123.44 (11)	C10—C9—H9	120.1
C9—C8—C14	120.75 (12)	C2—C3—C4	122.08 (12)
C9—C8—C7	119.01 (12)	C2—C3—H3	119.0
C14—C8—C7	120.24 (12)	C4—C3—H3	119.0
C5—C14—C8	119.17 (12)	C15—N1—C24	117.78 (12)
C5—C14—C13	120.74 (12)	C21—N2—C20	117.59 (12)
C8—C14—C13	120.09 (12)	C16—C17—C23	117.10 (12)
C11—C13—C14	117.33 (12)	C16—C17—C18	121.60 (12)
C11—C13—C2	125.99 (12)	C23—C17—C18	121.28 (12)
C14—C13—C2	116.68 (12)	C22—C18—C19	117.07 (12)
C10—C11—C13	120.61 (12)	C22—C18—C17	121.26 (12)
C10—C11—C12	114.95 (12)	C19—C18—C17	121.63 (12)
C13—C11—C12	124.19 (11)	C24—C23—C17	119.81 (13)
C3—C2—C13	120.43 (12)	C24—C23—H23	120.1
C3—C2—C1	115.54 (12)	C17—C23—H23	120.1
C13—C2—C1	123.96 (12)	C21—C22—C18	119.32 (13)
C4—C5—C14	120.61 (12)	C21—C22—H22	120.3
C4—C5—C6	119.51 (12)	C18—C22—H22	120.3
C14—C5—C6	119.84 (12)	N1—C15—C16	123.30 (13)
C11—C10—C9	121.30 (13)	N1—C15—H15	118.4
C11—C10—H10	119.3	C16—C15—H15	118.4
C9—C10—H10	119.3	N1—C24—C23	122.70 (13)
O7—C7—O6	116.81 (12)	N1—C24—H24	118.7
O7—C7—C8	125.34 (13)	C23—C24—H24	118.7
O6—C7—C8	117.76 (12)	C15—C16—C17	119.31 (13)
O5—C6—O6	116.80 (12)	C15—C16—H16	120.3
O5—C6—C5	125.81 (13)	C17—C16—H16	120.3
O6—C6—C5	117.39 (12)	N2—C21—C22	123.50 (13)
O3—C12—O4	126.01 (12)	N2—C21—H21	118.2
O3—C12—C11	122.11 (12)	C22—C21—H21	118.2
O4—C12—C11	111.69 (11)	N2—C20—C19	122.72 (14)
C5—C4—C3	119.14 (12)	N2—C20—H20	118.6
C5—C4—H4	120.4	C19—C20—H20	118.6
C3—C4—H4	120.4	C20—C19—C18	119.80 (14)
O1—C1—O2	126.31 (12)	C20—C19—H19	120.1
O1—C1—C2	122.40 (12)	C18—C19—H19	120.1
O2—C1—C2	111.18 (11)	C12—O4—H4A	109.5
C8—C9—C10	119.78 (12)	C1—O2—H2	109.5
C8—C9—H9	120.1		

### Hydrogen-bond geometry (Å, °)

**Table:**

*D*—H···*A*	*D*—H	H···*A*	*D*···*A*	*D*—H···*A*
O2—H2···N2^i^	0.84	1.77	2.594 (2)	167
O4—H4A···N1^ii^	0.84	1.74	2.573 (2)	171
C16—H16···O1^iii^	0.95	2.36	3.254 (2)	157
C22—H22···O3^iv^	0.95	2.57	3.425 (2)	150

Symmetry codes: (i) *x*, *y*−1, *z*−1; (ii) −*x*+1, −*y*+1, −*z*+1; (iii) *x*, *y*, *z*+1; (iv) −*x*+1, −*y*+2, −*z*+1.
